# DID COVID-19 INCREASE ICT USE AMONG MIDDLE-AGED AND OLDER JAPANESE?

**DOI:** 10.1093/geroni/igae098.2329

**Published:** 2024-12-31

**Authors:** Keiko Katagiri

**Affiliations:** Kobe University, Kobe, Hyogo, Japan

## Abstract

Information and Communication Technology (ICT) is permeating all facets of contemporary life, offering unprecedented convenience. Nonetheless, the digital schism is becoming increasingly pronounced, particularly among the older demographic. Instances where traditional social engagements are hindered, such as the inability to operate ATMs without ICT proficiency, may render older adults socially precarious. Amidst the COVID-19 pandemic, digital interactions supplanted face-to-face engagements. This study aims to scrutinize the surge in ICT adoption among middle-aged and older Japanese adults during the pandemic. An internet survey targeting individuals aged 50-69 residing in the Tokyo and Osaka metropolitan areas was conducted in March 2021. Participants exhibited a notable escalation in smartphone, tablet, personal computer, and WiFi utilization by 26.2%, 14.5%, 21.6%, and 24.3%, respectively. Moreover, 3.5%, 6.5%, 7.9%, and 34.8% of respondents commenced utilizing audio, images, videos, and online platforms, respectively, during the pandemic. Subsequently, multiple regression analyses were performed to discern factors influencing the adoption of these technologies. Participants manifested heightened usage of devices they were already familiar with. Furthermore, favorable evaluation of internet utility, female gender, non-employment status, and initiation of audiovisual media usage during the pandemic were positively associated with increased utilization of the aforementioned ICT devices. In conclusion, COVID-19 has played a pivotal role in mitigating the digital schism. Subsequent research endeavors should explore strategies to bolster middle-aged ICT engagement in the post-pandemic landscape.

